# When do parents and child health professionals agree on child’s psychosocial problems? Cross-sectional study on parent–child health professional dyads

**DOI:** 10.1186/s12888-016-0867-9

**Published:** 2016-05-19

**Authors:** Mathilde R. Crone, Elke Zeijl, Sijmen A. Reijneveld

**Affiliations:** Department of Public Health and Primary Care, Leiden University Medical Center, Public Health and Primary Care, P.O. Box 9600, 2300 RC Leiden, The Netherlands; Province of Gelderland, Department of Youth Care, P.O. Box 9090, 6800 GX Arnhem, The Netherlands; Department of Health Sciences, University Medical Center Groningen, University of Groningen, P.O. Box 196, 9700 AD Groningen, The Netherlands; TNO, Leiden, The Netherlands

**Keywords:** Identification, Mental health, Parental concerns, Agreement, Stressful events, Vulnerability, Preschool child, Child

## Abstract

**Background:**

About one third of all parents have concerns about their child’s psychosocial development. Agreement between child health professionals (CHPs) and parents about such concerns may improve treatment adherence and outcomes. This study investigates which child, parenting and/or environmental stressors are associated with (dis)agreement in concerns regarding psychosocial problems in children, in parent-CHP dyads.

**Methods:**

During routine child health assessments, data were collected from a sample of children aged 14 months to 12 years (*n* = 3,870). CHPs registered the psychosocial problems that they identified, and parents reported their concerns. Child psychosocial stressors were measured with the ITSEA/CBCL, and the child’s history of psychosocial problems. Environmental stressors referred to stressful family/contextual situations in the past year, and parenting stressors to perceived parenting efficacy.

**Results:**

The CHPs and parents disagreed on 36.4 % of the children. CHPs based their identification of problems mainly on children’s history of past problem (OR = 5.85, 95 % CI = 4.74–7.22). Parental concerns were most likely in case of an increased ITSEA/CBCL score (OR = 7.69, CI = 5.39–10.97). CHP-parent agreement was more likely in case of a combination of child psychosocial, parenting and environmental stressors (OR = 35.58, CI = 24.11–52.48). Parental concerns *not* confirmed by the CHP were associated with higher educated parents, originating from an industrialized country, and younger children. The CHP-identified problems *not* confirmed by parental concerns were associated with older children.

**Conclusion:**

Agreement between CHPs and parents is associated with a co-occurrence of child, parenting and environmental stressors. Improved agreement between CHP and parents will increase the likelihood of shared decision-making regarding follow-up care and compliance with advice.

## What is new?

Children requiring assistance according to both the parents and child health professionals (CHPs) more often had child stressors in combination with parenting and/or environmental stressors. Disagreements between CHPs and parents were due to 1) parents being more worried about preschool children and current problems, and 2) CHPs identifying more problems in school-aged children, and chronic or persisting problems.

## Background

Psychosocial problems are highly prevalent among children [1], have a negative and often persistent influence on children’s daily lives, and may lead to adverse outcomes later on [2–5]. Early identification of psychosocial problems is an important first step in preventing severe problems later in life, and may improve the child’s prognosis [6].

In the Netherlands, community pediatric services are important for the early identification of psychosocial problems in children. Comparable to professionals working in community pediatric services in the USA, child health professionals (CHPs) working in Dutch services routinely offer preventive care to all children aged 0–19 years. About 80–90 % of all children undergo about ten CHP assessments until the age of 4 years, and about three assessments during their school career.

CHPs identify psychosocial problems in about 25 % of the children that visit them [7]. However, the sensitivity of CHPs in identifying children with behavioral and/or emotional problems based on an independent assessment (e.g. diagnostic interviews or validated screening instruments) is relatively low [7, 8]. CHP identification is generally based on information provided by parents. About 30 % of parents have minor to severe concerns regarding the psychosocial development of their child [9, 10]. These concerns are significantly associated with elevated problem scores on behavioral screening tools [11–13]. Therefore, if these concerns are disclosed, the likelihood of CHPs identifying problems in these children will be much higher [6, 14, 15]. However, even when disclosed, CHPs do not always confirm all parental concerns and, vice versa, not all parents agree with all the CHP-identified problems [10]. This discrepancy between CHP and parents hampers appropriate and effective care [16].

Few studies have simultaneously investigated professionals’ and parents’ viewpoints to explore potential reasons for this discrepancy [14, 17]. Studies examining agreement mainly focus on agreement based on the assessment instruments completed by parents, adolescents and teachers; [18] generally, they do not examine the agreement between parents and professionals regarding concerns. In addition, an increased score on these assessment instruments does not imply the presence of parental or CHP concerns and, conversely, a score in the normal range does not imply an absence of concerns [10, 15, 19].

The present study used data of parents-CHP dyads to explore the agreement between the identified psychosocial problems in children and the factors influencing this agreement. In line with assessment frameworks of child development, stressors related to the child, parents, and family and environmental context, were studied [20]. The aim was to explore whether these stressors are associated with parent-CHP (dis)agreement and whether a combination of these stressors is more strongly related to agreement.

## Methods

### Study sample

Between October 2002 and June 2003 we recruited a national sample of community pediatric services: in total 25 services participated. Ten services had preventive health assessments with children aged 0–4 years and 15 services with children aged 4–12 years. Each service was asked to interview a random sample of at least 100 children that visited the CHP for a routine preventive health assessment in four age groups: i) 14 months, ii) 3 years 9 months, iii) 5/6 years, and iv) 8–12 years.

From the 5,611 eligible children, 85.1 % (*n* = 4,776) participated; the main reason for non-response was refusal to participate. For 447 children the parents did not complete the questionnaire, whereas the CHP did; these children more often had mothers with a lower educational level (55.0 % for children with CHP data only versus 38.2 % for children with both CHP and parent data), were from a non-industrialized country (36.0 % versus 14.0 %), came from a one-parent household (14.4 % versus 7.5 %), and were younger (48.1 % versus 36.1 % aged ≤ 4 years). There were no differences in the CHP-identified psychosocial problems. For 162 children no data were available from the CHP whereas the parents had completed the questionnaire; these children did not differ from children with data from both informants regarding the reported concerns of the parents (no background characteristics available).

Included in the study were children for whom parents and CHPs had filled out the questionnaire (*n* = 4,168). For the analyses, data were used only from those children who were not yet being treated for psychosocial problems (95 %) and who had no missing answers on the variables under investigation. This resulted in a total sample of 3,870 children.

### Procedure

Data were collected as part of the preventive health assessment; a questionnaire was mailed to the parents together with the invitation to attend the assessment. Parents completed the questionnaire and returned it to the researchers in a sealed envelope (CHPs were not permitted to view the questionnaire): 84 % of the parents completed the questionnaire prior to the assessment and 16 % completed it afterwards.

After each child’s physical examination, during a standardized interview with the parents the CHP obtained information on the child’s/family background and on mental health history. After this interview and the health assessment of the child, CHPs registered the psychosocial problems they had identified (all CHPs received prior instruction from the research team about how to register the socio-economic characteristics and the identified psychosocial problems). In a separate questionnaire, CHPs were asked about their general use of screening tools to identify psychosocial problems.

Translations of the Child Behavior CheckList (CBCL) and additional questionnaires were provided in English, Arabic and Turkish. Also, Turkish and Moroccan interpreters were available to assist Turkish and Moroccan parents in completing the questionnaire. Parents from Surinam and the Netherlands Antilles (former Dutch colonies) generally speak the Dutch language. Bias due to interpretation of the questionnaire by the translators was prevented as far as possible by discussing the meaning of each item on the questionnaires with them.

### Measures

*CHP-parent agreement* concerned the identification of problems by the CHP, and the concerns of parents about the psychosocial problems of the child.

For the *CHP-identified psychosocial problems,* the CHP filled out the following question after each assessment: ‘Does the child have a psychosocial problem, at this moment?’ (yes, no) and scored the type of identified problem(s) on a pre-coded list. The CHP was instructed to code problems such as sleeping or eating problems as problems **only** when they suspected that they were related to psychosocial problems. These questions were comparable to the ones used in an earlier study [7].

*Parental concerns* about their child’s psychosocial problems were obtained by a parental concerns questionnaire regarding social, behavioral and/or emotional problems of their child in the past 12 months, for which they felt the child needed professional assistance. The responses were ‘no concerns’, ‘some concerns’ or ‘serious concerns’. Parents were categorized as: 1) either having no concerns, or 2) having some to severe concerns about at least one of the problems.

*Agreement* between CHP-identified problems and parental concerns was categorized as: 1) no CHP-identified problems and no parental concerns, 2) no CHP-identified problems, but parental concerns, 3) CHP-identified problems, but no parental concerns, and 4) both CHP-identified problems and parental concerns.

*Child psychosocial stressors* referred to an increased psychosocial total problems score of the child, and a history of psychosocial problems. The *psychosocial total problems score* was measured by the Infant Toddler Social and Emotional Assessment (ITSEA), and the CBCL for ages 1.5–5 years and ages 6–18 years [21–24]. These instruments assessed the parent’s report on the child’s behavioral and emotional symptoms during the preceding 6 months. In the group of infants aged 14 months, parents completed the ITSEA: a well-validated questionnaire for parents with a child aged 12–36 months, consisting of 166 items measuring 17 syndrome scales [23, 24]. For the present study, parents filled out the items of 12 syndrome scales (activity/impulsivity, aggressive behavior, depressive/withdrawn, general anxiety, separation distress, inhibition to novelty, sleeping problems, negative emotionality, eating problems, tactile sensitivity, and attention problems). They represent three broadband groups (Externalizing, Internalizing and Dysregulation) and one total problems scale, and has a good factor structure and acceptable internal consistency [25]. Parents of children aged 3 years 9 months, and 5/6 years, completed the CBCL 1.5–5, which consists of 100 problem items. Parents of 8–12 year old children completed the CBCL 6–18 consisting of 113 problem items [21, 22]. In the present study, only the Total Problems scores of the ITSEA and the CBCL were used. Children were allocated to a normal or a clinical range based on the cut-off points of the USA normative sample which, according to the CBCL developers, are also valid for Dutch children [26].

Regarding *history of problems,* the CHP asked parents whether the child had received earlier assistance from a professional because of psychosocial problems (no/yes). The CHP also indicated whether, in a former CHP contact, psychosocial problems were registered in the child’s file (no/yes). Children were categorized as: 1) having no history of psychosocial problems, or 2) having a history.

*Parenting stressors* were measured by 10 statements on parenting efficacy (with answers ranging from 1 = totally disagree to 4 = totally agree): i.e. whether parents experience parenting as a burden, are satisfied about how they parent, and always know what to do in various parenting situations. This standardized measure is frequently used by the Netherlands Institute for Social Research to assess parents’ experience with parenting, and is significantly correlated with parenting styles and the need of parents for parenting assistance [27]. A Cronbach’s α of 0.71 indicated a reasonable reliability. A sum score was calculated for each parent (scale from 1 to 4). As the distribution was skewed to the right, the sum score was dichotomized into 1) parents with sufficient parenting efficacy (score ≥ 3), and 2) parents with less parenting efficacy (score < 3).

*Family and environmental stressors* were asked during the interview between the parents and the CHP, and concerned several pre-formulated family and environmental life events of the child during the past 12 months based on the Coddington Life Event Questionnaire; this questionnaire has been validated for the Netherlands and several other countries [28–30]. Events ranged from the birth of a brother/sister, divorce, illness of family members, death of a family member, to unemployment and low income (below or at the poverty line). Children were categorized as 1) having a maximum of one stressful situation, or 2) more than one.

#### Child, family and CHP background characteristics

Child and family background assessed by the CHP were: gender, age, ethnicity, and highest educational level. *Ethnicity* was based on the country of origin of the biological parents of the children. Children were coded as originating from an industrialized country when both parents were indigenous Dutch, or born in another country that resembles the Dutch population in socio-economic status and cultural position (Statistics Netherlands: www.cbs.nl): defined as countries that are a member of the Organisation for Economic Co-operation and Development (OECD), with the exception of Turkey. Children were coded as originating from a non-industrialized country when at least one of the parents was born in Turkey, or in a country that is not a member of the OECD. *Educational level* was determined by the highest level of education completed by the parents and classified according to the International Standard Classification of Education: 1) low level, no, primary or lower secondary education; 2) average level, upper secondary education or post-secondary non-tertiary education; and 3) high level, recognized tertiary education [31]. As the identification of problems by the CHP is partly influenced by the use of screening tools and is likely to have an impact on the CHP-parent agreement, the CHPs were asked about their *use of screening questionnaires* to identify psychosocial problems during routine preventive health assessments: 1) never, 2) when indicated, 3) for every child.

### Statistical analysis

First, we examined the characteristics of children and CHP-parent agreement (frequencies and Cohen’s Kappa), and the association between these characteristics and the variables of agreement, i.e. identification and concerns. Univariate and multivariate multilevel logistic regression analyses were performed to assess whether stressors and background characteristics were significantly associated with CHP identification and with parental concerns. Then, these logistic analyses were repeated including (instead of the individual factors) the combination of child, parenting and context stressors in the model, i.e. 1) no child, parenting or environmental stressors, 2) no child stressors, but parenting or environmental stressors, 3) child stressors, but no parenting or environmental stressors, 4) child and parenting or environmental stressors. Multinomial regression analyses were conducted to determine whether the combination of these groups of stressors led to more parent-CHP agreement than the separate groups. All data were analyzed with the SPSS.

## Results

In total, 63 % of the participating children were aged 5–12 years, about 50 % were girls and 80 % were of Dutch origin or from an industrialized country (Table 1).

**Table 1:** Child and family characteristics associated with CHP-identified psychosocial problems and parental concerns among children aged 0–12 years

	Total	CHP identified psychosocial problems		Parental concerns	
	% n, *n* = 3,870	No % n, *n* = 3,193	Yes % n, *n* = 677	No % n, *n* = 2,388	Yes % n, *n* = 1,482
Total Sample		82.5	17.5	61.7	38.3
Child: CBCL/ITSEA problem score			***		***
No clinical score	95.3	97.0	87.4	98.7	89.9
Clinical score	4.7	3.0	12.6	1.3	10.1
Child: history of problems			***		***
No	84.3	90.2	56.3	88.9	76.7
Yes	15.7	9.8	43.7	11.1	23.3
Environmental: Life events			**		***
No	88.9	89.9	84.2	90.5	86.2
Yes	11.1	10.1	15.8	9.5	13.8
Parenting: Parenting efficacy			***		***
Efficacy	77.1	79.0	68.1	82.5	68.5
Low efficacy	22.9	21.0	31.9	17.5	31.5
Gender			***		
Girl	51.1	52.6	44.0	52.0	49.7
Boy	48.9	47.4	56.0	48.0	50.3
Ethnicity			*		
Industrialized country	81.3	82.0	78.0	79.9	80.5
Non-Industrialized country	19.7	18.0	22.0	20.1	19.4
Age			***		***
14 months	18.8	20.7	9.6	18.1	19.8
3 years 9 months	18.2	18.9	14.6	16.3	21.3
5/6 years	34.3	31.3	48.2	33.6	35.4
8–12 years	28.8	29.0	27.6	32.0	23.5
Educational level			***		
Low	38.0	36.5	45.2	39.3	35.8
Average	36.0	36.5	33.4	35.3	37.2
High	24.3	25.2	19.8	23.6	25.4
Unknown	1.7	1.7	1.6	1.7	1.6
CHP’s use of screening instruments			***		***
Never	83.0	83.9	78.9	83.0	83.0
When indicated	11,3	11.0	12.4	11.1	11.5
At each assessment	5.7	5.1	8.7	5.9	5.5
Combination child, parenting, environmental stressors			***		***
Not in child and parenting/context	60.1	65.5	34.3	68.6	46.4
Not in child but in parenting/context	21.2	22.3	16.2	19.3	24.3
In child, but not in parenting/context	10.1	6.9	25.4	7.7	14.1
Both in child and parenting/context	8.6	5.3	24.1	4.4	15.2

*Differences in child and family characteristics between children with and without CHP-identified psychosocial problems and between parents with and without concerns regarding development of the child:* = *p* < 0.05; ** = *p* < 0.01, *** = *p* < 0.001

In 17.5 % of the children, the CHP identified psychosocial problems and in 38.5%parents had concerns about their child’s psychosocial development for which they felt professional assistance is needed. CHPs most frequently identified internalizing problems (6.7 %), followed by other developmental problems (5.8 %), and externalizing problems (5.0 %). Parents most frequently had concerns about the child’s behavioral (23.1 %) and emotional development (14.0 %). In 63.6 % of the children, parents and CHPs agreed as to whether or not the child had a problem. Children of parents that completed the questionnaire after the CHP visit did not differ in agreement rate from those who completed the questionnaire in advance, i.e. 64.4 % versus 63.4 %.

In case of disagreements, these most often referred to children for whom parents had concerns that were not confirmed by the CHP (Table 2). In particular, parental concerns regarding the behavioral development of the child were less frequently identified by the CHP. This was also reflected by the lower inter-rater reliability between CHPs and parents regarding behavioral problems (Kappa = .066; standard error = .014). The inter-rater reliability for internalizing problems was .109 (.019), and for social problems .144 (.025). Overall the inter-rater reliability scores indicated that the agreement between parents and CHPs was just slightly higher than could be expected from chance alone.

**Table 2:** Child and family characteristics associated with (dis)agreement between CHP and parents on psychosocial problems in children aged 0–12 years

	CHP-parent agree on child having **no** problems %n, *n* = 2,086	Parents have concerns but CHP identifies no problems %n, *n* = 1,107	Parents have no concerns, CHP identifies problems %n, *n* = 302	Parents and CHP agree that child has problems % n, *n* = 375
Total sample	53.9	28.6	7.8	9.7
Child: CBCL/ITSEA problem score	***			
No clinical score	99.1	93.0	95.7	80.8
Clinical score	0.9	7.0	4.3	19.2
Child: history of problems	***			
No	92.4	86.1	65.2	49.1
Yes	7.6	13.9	34.8	50.9
Environmental: Life events	***			
No	91.4	86.9	84.1	84.3
Yes	8.6	13.1	15.9	15.7
Parenting: Parenting efficacy	***			
Higher efficacy	83.0	71.5	78.5	59.7
Lower efficacy	17.0	28.5	21.5	40.3
Gender	**			
Girl	53.0	52.0	45.7	42.7
Boy	47.0	48.0	54.3	57.3
Ethnicity				
Industrialized country	81.7	82.5	77.2	78.7
Non-Industrialized country	18.3	17.5	22.8	21.3
Age	***			
14 months	19.6	22.8	7.9	10.9
3 years 9 months	16.9	22.9	12.3	16.5
5/6 years	31.4	31.3	48.7	47.7
8–12 years	32.1	23.1	31.1	24.8
Educational level	**			
Low	38.1	33.3	47.7	43.2
Average	36.0	37.8	30.8	35.5
High	24.2	27.2	19.5	20.0
Unknown	1.7	1.7	2.0	1.3
CHP’s use of screening instruments	**			
Never	83.6	84.5	79.1	78.7
When indicated	11.1	10.9	11.3	13.3
At each assessment	5.4	4.6	9.6	8.0
Combination child, parenting, environmental stressors	***			
Not in child and parenting/context	71.8	53.7	46.0	24.8
Not in child but in parenting/context	19.8	27.1	16.6	16.0
In child, but not in parenting/context	5.4	9.8	23.5	26.9
Both in child and parenting/context	3.1	9.5	13.9	32.3

*Differences in child and family characteristics between categories of agreement between CHP and parents the child:* = *p* < 0.05; ** = *p* < 0.01, *** = *p* < 0.001

Table 3 shows that (univariately) an increased CBCL/ITSEA score, a history of problems, exposure to environmental stressors, lower parenting efficacy, school-aged children, boys, children from a non-industrialized country, lower educational level, and the use of screening instruments by CHPs, were significantly associated with CHP identification of psychosocial problems. In a multivariate regression analysis all these factors (except environmental stressors and ethnicity) remained significant predictors of CHP identification. A history of psychosocial problems was the strongest predictor of problem identification by CHPs. CHP-identified problems were most frequently observed in children who showed stressors in both the child, and the parenting and/or environmental domain. Children exposed only to parenting and/or environmental stressors were not identified with psychosocial problems more often than children without such stressors.

**Table 3:** Child/family characteristics associated with CHP-identified psychosocial problems or parental concerns in children aged 0–12 years

	CHP identifies problems			Parents have concerns		
	% CHP identified problems	Unadjusted OR (95 % CI) *n* = 3,870	Adjusted OR^#^ (95 % CI) *n* = 3,870	% parental concern	Unadjusted OR (95 % CI) *n* = 3,870	Adjusted OR^#^ (95 % CI) *n* = 3,870
Child: CBCL/ITSEA problem score						
No clinical score$	16.0	1	1	36.0	1	1
Clinical score	47.2	**4.66 (3.37–6.44)**	**3.43 (2.41–4.89)**	82.8	**8.63 (6.17–12.07)**	**7.69 (5.39–10.97)**
Child: history of problems						
No$	11.7	1	1	34.9	1	1
Yes	48.6	**6.56 (5.29–8.14)**	**5.85 (4.75–7.21)**	56.7	**2.47 (2.05–2.98)**	**2.28 (1.91–2.73)**
Environmental: life events						
No$	16.6	1	1	37.2	1	1
Yes	24.8	**1.60 (1.25–2.05)**	1.20 (0.92–1.59)	47.3	**1.54 (1.23–1.93)**	**1.55 (1.21–1.99)**
Parenting: Parenting efficacy						
Higher efficacy$	15.4	1	1	34.0	1	1
Lower efficacy	24.4	**1.73 (1.43–2.09)**	**1.28 (1.05–1.56)**	52.7	**2.17 (1.88–2.51)**	**1.92 (1.65–2.23)**
Gender						
Girl$	15.1	1	1	37.2	1	1
Boy	20.0	**1.38 (1.18–1.61)**	**1.21 (1.02–1.43)**	39.5	1.10 (0.96–1.25)	0.98 (0.85–1.12)
Ethnicity						
Industrialized country$	16.8	1	1	38.4	1	1
Non-Industrialized country	20.6	**1.27 (1.18–1.61)**	0.87 (0.66–1.14)	37.8	0.99 (0.83–1.18)	**0.78 (0.64–0.94)**
Age						
14 months$	9.0	1	1	40.4	1	1
3 years 9 months	14,1	**1.67 (1.04–2.68)**	1.39 (0.89–2.16)	44.7	1.20 (0.97–1.48)	1.14 (0.91–1.42)
5/6 years	24.6	**3.20 (2.03–5.04)**	**2.49 (1.55–4.00)**	39.6	0.96 (0.77–1.21)	0.90 (0.71–1.14)
8–12 years	16.8	**2.40 (1.53–3.77)**	**1.61 (1.00–2.58)**	31.4	**0.67 (0.53–0.85)**	**0.60 (0.47–0.76)**
Educational level						
Low$	20.8	1	1	36.1	1	1
Average	16.2	**0.72 (0.59–0.88)**	0.85 (0.69–1.05)	39.5	1.04 (0.66–1.64)	**1.25 (1.05–1.50)**
High	14.3	**0.56 (0.44–0.72)**	**0.70 (0.53–0.91)**	40.0	1.16 (0.96–1.40)	**1.39 (1.13–1.71)**
Unknown	16.9	0.74 (0.41–1.34)	0.62 (0.35–1.08)	36.9	1.14 (0.98–1.34)	**1.10 (0,66–1.82)**
CHP’s use of screening instruments						
Never$	16.6	1	1	38.3	1	1
When indicated	19.3	1.16 (0.81–1.66)	**1.53 (1.10–2.14)**	39.2	1.06 (0.75–1.50)	1.00 (0.73–1.37)
At each assessment	26.6	**1.85 (1.27–2.70)**	1.38 (0.86–2.22)	36.5	0.93 (0.71–1.22)	0.90 (0.72–1.14)
Combination child, parenting, environmental stressors			&			&
Not in child and parenting/context$	10.0	**1**	1	29.6	**1**	**1**
Not in child but in parenting/context	13.4	**1.33 (1.01–1.76)**	1.28 (0.96–1.71)	43.8	**1.88 (1.59–2.22)**	**2.11 (1.77–2.51)**
In child, but not in parenting/context	43.9	**6.40 (4.90–8.38)**	**6.20 (4.72–8.13)**	53.3	**2.78 (2.21–3.48)**	**3.11 (2.47–3.91)**
Both in child and parenting/context	49.1	**8.16 (6.22–10.70)**	**7.84 (5.85–10.51)**	68.1	**5.20 (4.26–6.36)**	**6.24 (5.04–7.73)**

# Adjusted for other parent, child, environmental stressors and child, family, and CHP background characteristics. $ = Reference group

**Bold**: significant ORs, *p* < 0.05

& adjusted for child, family, CHP background characteristics

Parental concerns about their child’s psychosocial development were more likely when the child had an increased CBCL/ITSEA score, a history of problems, environmental stressors, less effective parents, and parents with a higher educational level. The strongest predictor was an increased score on the CBCL/ITSEA. Fewer concerns were reported by parents of children aged 8–12 years and by parents from non-industrialized countries. Similar to CHP-identified problems, parental concerns were most likely to occur in children with child stressors combined with parenting and/or environment stressors.

The agreement between parents and CHPs on the presence of problems was strongly associated with a combination of child, and parenting and/or environmental stressors. When parents had concerns *not* confirmed by the CHP, this association was weaker. These non-confirmed children were relatively more often exposed to only parenting and/or environmental stressors, while no child psychosocial stressors were reported. Also, these children were more often from higher educated families, and less often aged 8–12 years or from non-industrialized countries. In contrast, children with CHPs identified problems *not* confirmed by parental concerns more often were aged 8–12 years (Table 4).

**Table 4:** Multinomial regression analysis comparing CHP-parent agreement on the child having no problems with CHP-parent agreement on the child having problems, and CHP-parent disagreement with the child having problems

	Reference: CHP-parent agreement on the child having **no** psychosocial problems (*n* = 2,086)			
	% children on which CHP and parent agree there are problems	Parents have concerns but CHP identifies no problems OR (95 % CI)# *n* = 1,107	Parents have no concerns but CHP identifies problems OR (95 % CI)# *n* = 302	Parents and CHP agree that child has problems OR (95 % CI)# *n* = 375
Child: CBCL/ITSEA total problem score				
No clinical score$	8.2	1	1	1
Clinical score	40.0	**8.57 (5.03–14.61)**	**4.44 (2.10–9.37)**	**22.38 (12.56–39.88)**
Child: history of problems				
No$	5.6	1	1	1
Yes	31.4	**1.93 (1.51–2.46)**	**5.78 (4.31–7.76)**	**11.26 (8.53–14.87)**
Environmental: Stressful situations				
No$	9.2	1	1	1
Yes	13.7	**1.77 (1.36–2.30)**	**1.55 (1.05–2.27)**	**1.50 (1.03–2.20)**
Parenting: Parenting efficacy				
Higher efficacy$	7.5	1	1	1
Lower efficacy	17.0	**1.82 (1.52–2.19)**	1.11 (0.81–1.52)	**2.45 (1.87–3.20)**
Gender				
Girl$	8.1	1	1	1
Boy	11.4	0.96 (0.83–1.12)	1.25 (0.97–1.61)	1.20 (0.93–1.53)
Ethnicity				
Industrialized country$	9.4	1	1	1
Non-Industrialized country	11.0	**0.78 (0.62–0.98)**	0.86 (0.61–1.21)	**0.66 (0.47–0.93)**
Age				
14 months$	5.6	1	1	1
3 years 9 months	8.8	1.15 (0.91–1.44)	1.66 (0.97–2.84)	1.46 (0.92–2.31)
5/6 years	13.5	0.84 (0.68–1.04)	**3.25 (2.04–5.17)**	**2.55 (1.71–3.82)**
8–12 years	8.4	**0.58 (0.46–0.73)**	**1.83 (1.13–2.96)**	1.00 (0.65–1.55)
Educational level				
Low$	11.0	1	1	1
Average	9.5	**1.25 (1.04–1.51)**	0.85 (0.63–1.14)	1.14 (0.85–1.53)
High	8.0	**1.43 (1.17–1.74)**	0.76 (0.54–1.07)	1.05 (0.74–1.47)
Unknown	7.7	1.24 (0.68–2.25)	0.81 (0.32–2.05)	0.60 (0.21–1.74)
CHP’s use of screening instruments				
Never$	9.2	1	1	1
When indicated	11.5	0.93 (0.73–1.19)	1.35 (0.90–2.02)	**1.61 (1.10–2.35)**
At each assessment	13.5	0.91 ((0.64–1.30)	1.27 (0.80–2.01)	1.03 (0.64–1.67)
Combination child, parent, environmental stressors		&	&	&
Not in child and parenting/context$	4.0	1	1	1
Not in child but in parenting/context	7.3	**2.08 (1.73–2.51)**	1.21 (0.85–1.73)	**2.51 (1.77–3.58)**
In child, but not in parenting/context	25.8	**2.76 (2.07–3.67)**	**6.26 (4.39–8.91)**	**15.39 (10.84–21.86)**
Both in child and parenting/context	36.4	**5.05 (3.61–7.07)**	**6.67 (4.28–10.40)**	**35.58 (24.11–52.48)**

$ = Reference group

# Adjusted for other parent, child, environmental stressors and child, family, and CHP background characteristics

**Bold**: significant ORs, *p* < 0.05

& adjusted for child, family, CHP background characteristics

## Discussion

This study indicates that there are disagreements between CHP-identified problems and parental concerns about the child’s psychosocial problems, in one of every three children. Mutual agreement on the need for professional assistance was more likely to occur if the child had just started school, showed child stressors due to the manifestation of symptoms, a history of psychosocial problems in combination with environmental stressors, and lower parenting efficacy. Parental concerns most often concerned worries about the child’s behavioral development, and were most likely to be present in the case of a clinical ITSEA/CBCL score, and in toddlers. However, concerns were frequently not confirmed by the CHP in younger children from industrialized countries and in higher educated families. Children identified by CHPs more often had a history of problems and tended to be school-aged. In children aged 8–12 years, CHPs more often perceived problems, whereas their parents had fewer or no concerns.

In line with our expectations, a combination of child, parenting and environmental stressors increases the agreement between professional and parent. Problems are more likely to be apparent in these children, and studies on either parents or professionals show that both recognize severe problems more easily [13, 32]. In contrast, when CHP and parents disagree, stressors co-occur less frequently and nearly 50 % reported no stressors. About 25 % of the children with parental concerns not confirmed by the CHP had parenting and/or environmental stressors but no child stressors.

Child stressors differ to some extent between parents and CHPs. CHPs seem to focus more on history or the persistence of problems, whereas current problems (as expressed by the CBCL/ITSEA score) appear to disturb parents the most, which is also reflected in lower parenting efficacy. In particular, this parental burden and appraisal of problems relate to the help-seeking behavior of parents and child mental health services use [33, 34]. However, differences in perspectives on child problems can also be caused by reporter bias: e.g. in the present study, parents completed the CBCL/ITSEA and the parenting efficacy scale, whereas the CHPs registered the history of problems and life events. Nevertheless, the CHP registration and identification was based on parental reports (during the interviews), implying less risk of reporter bias with regard to the history of problems and life events.

Generally, parents with younger children tend to have more concerns, whereas CHPs do not always confirm these concerns [9, 10, 35]. This may be related to the finding that the accuracy of parental concerns for detecting mental health problems is lower when the child is younger [9]. Parents with concerns sometimes have misconceptions regarding what is the ‘normal’ development for a child [6, 36]. The present study suggests that these unconfirmed parental concerns might also express other problems related to parenting ability and environmental stressors; this implies that the CHP’s decision not to recognize them as child psychosocial problems might be adequate. Also, during the encounter with the CHP, parents with younger children may less often express their concerns compared with parents of older children, because they are unaccustomed to asking for help regarding the mental health problems of their child, or believe that it is too early to detect psychosocial problems [6, 37]. However, CHPs may not always be accurate in recognizing and discussing these concerns, even when disclosed by the parents.

In contrast to our findings, Blanchard et al. found that parents were more often concerned about problems in school-aged children than in younger children [38]. The discrepancy between the latter study and ours might be related to differences in collecting data on parental concerns. Their questions differed between younger and older children and also referred to worries about learning difficulties; these were prominent concerns in their school-aged children but were not included as concerns in the present study.

Similar to Brugman et al. we found that CHP identification was most prevalent in children aged 5/6 years who had just started elementary school [7]. Their problems may reflect the psychosocial problems that a child encounters when entering the school setting. Moreover, schools are frequently the first setting in which children can be observed among their peers by both professionals and parents. This might explain the finding that parents and CHPs were more likely to agree on problems related to children in this age group.

Parents of children aged 8–12 years with CHP-identified psychosocial problems reported fewer concerns. Older children are increasingly able to express their own concerns and thereby become an essential informant for the CHP. This may clarify some discrepancies between parents and CHPs in older children. Another explanation may be that, as the child grows older, parents have become more skilled in coping with the psychosocial problems of their child, leading to a reduction of parental concerns needing professional assistance. This may apply in particular to concerns about behavioral development, which were less often reported by parents of this age group (15 % versus 21 % to 32 % in the younger age groups).

### Strengths and limitations

Strengths of the study are the size of the community-based sample, and the availability of data from two types of informants. A limitation is that the non-response was higher for parents from non-industrialized countries and from one-parent households. However, because the percentage of CHP-identified problems did not differ between the response and non-response groups, the impact on the present findings seems limited. Another limitation is the use of parental self-reports to assess the child’s psychosocial problem score, parental concerns and parenting efficacy. However, we used valid, reliable and frequently used questionnaires (i.e. the CBCL and ITSEA), which makes bias less likely [21–24]. A third limitation is that we did not obtain information on the process of the visit itself, i.e. whether or not parents discussed any problems with the CHP that led to a different evaluation. Although about 16 % of the parents ‘forgot’ to complete the questionnaire before the visit and did it afterwards, and their level of agreement and presence of stressors did not differ from those parents who completed the questionnaire in advance.

We only included those parental concerns for which parents felt that they needed professional assistance; the present rates do not reflect all parents’ concerns regarding their child’s psychosocial development. This may have led to an underrepresentation of concerns of parents from non-industrialized countries, as they are less willing to talk about problems outside the direct family and, consequently, appear to need less professional assistance [39].

CHPs were not able to check the CBCL/ITSEA filled out by the parents. Therefore, it remains unknown what the agreement would have been had the CHPs been able to view the CBCL/ITSEA score. Nevertheless, a discrepancy between CHP and parents is still likely (even if they had viewed the CBCL/ITSEA), as professionals often do not use the recommended cut-off points of such instruments to guide their decisions [19]. Moreover, the sensitivity of CHPs in identifying children with problems on an assessment instrument when using a screening tool does not differ from that when they did not use such a tool [40]. In addition, the CHPs received instruction on interviewing parents about their socio-economic status and the mental health history of their child. Although the study did not provide training in the identification of psychosocial problems, conducting these interviews during the preventive health assessment may have had an impact on the CHPs’ awareness and recognition of problems.

## Conclusions

In the present study, the majority of children, parents and CHPs agree on whether or not the child has psychosocial problems. Nevertheless, there is disagreement regarding about one third of all children, mostly due to parental concerns not being confirmed by the CHP. Even when using screening instruments in the identification process, there will be some disagreement between parents and CHPs [19]. Future research should explore the parent-CHP interaction to further elucidate the decision-making process regarding the identification of psychosocial problems in children. In addition, the longer-term impact on uptake of treatment and psychosocial problems should be assessed to determine the efficacy of CHP-identified psychosocial problems.
